# Matrine treatment reduces retinal ganglion cell apoptosis in experimental optic neuritis

**DOI:** 10.1038/s41598-021-89086-7

**Published:** 2021-05-04

**Authors:** Jian Kang, Shuqing Liu, Yifan Song, Yaojuan Chu, Mengru Wang, Yamin Shi, Fengyan Zhang, Lin Zhu

**Affiliations:** 1 Department of Pharmacy, The First Affiliated Hospital of Zhengzhou University, Zhengzhou, Henan China; 2 Department of Ophthalmology, Beijing Key Laboratory for Restoration of Injured Ocular Nerve, Peking University Third Hospital, Beijing, China; 3 Department of Chinese Medicine, The First Affiliated Hospital of Zhengzhou University, Zhengzhou, Henan China; 4 Department of Ophthalmology, The First Affiliated Hospital of Zhengzhou University, Zhengzhou, Henan China

**Keywords:** Immunology, Molecular biology

## Abstract

Inflammatory demyelination and axonal injury of the optic nerve are hallmarks of optic neuritis (ON), which often occurs in multiple sclerosis and is a major cause of visual disturbance in young adults. Although a high dose of corticosteroids can promote visual recovery, it cannot prevent permanent neuronal damage. Novel and effective therapies are thus required. Given the recently defined capacity of matrine (MAT), a quinolizidine alkaloid derived from the herb Radix Sophorae flavescens, in immunomodulation and neuroprotection, we tested in this study the effect of matrine on rats with experimental autoimmune encephalomyelitis, an animal model of multiple sclerosis. MAT administration, started at disease onset, significantly suppressed optic nerve infiltration and demyelination, with reduced numbers of Iba1^+^ macrophages/microglia and CD4^+^ T cells, compared to those from vehicle-treated rats. Increased expression of neurofilaments, an axon marker, reduced numbers of apoptosis in retinal ganglion cells (RGCs). Moreover, MAT treatment promoted Akt phosphorylation and shifted the Bcl-2/Bax ratio back towards an antiapoptotic one, which could be a mechanism for its therapeutic effect in the ON model. Taken as a whole, our results demonstrate that MAT attenuated inflammation, demyelination and axonal loss in the optic nerve, and protected RGCs from inflammation-induced cell death. MAT may therefore have potential as a novel treatment for this disease that may result in blindness.

## Introduction

Optic neuritis (ON) is a disease that affects young adults ranging from 18 to 45 years of age, and also children as young as 4, which involves primary inflammation, demyelination, and axonal injury in the optic nerve^1–3^. The annual incidence of ON is approximately 5 in 100,000, with a prevalence estimated to be 115 in 100,000. It can be clinically isolated or can develop as one of the manifestations of multiple sclerosis (MS)^4,5^. In 15–20% of individuals who eventually develop MS, ON is their first sign of disease. An acute, self-limited episode of optic nerve inflammation results in demyelination, accompanied by temporary or permanent loss of vision^6,7^.

Retinal ganglion cells (RGCs), the projection neurons of the eye, undergo apoptosis with ON in the experimental autoimmune encephalomyelitis (EAE) model, and a significant loss of RGCs due to apoptosis has been demonstrated after optic nerve injury^8^. Once the optic nerve is damaged, RGCs will die and axons will fail to regenerate, leading to traumatic or ischemic nerve injury or degenerative conditions^9^. The death of RGCs has been considered the main cause of vision loss after an episode of ON^10,11^. In the animal model of relapsing/remitting EAE, RGC apoptosis begins within a few days after onset of optic nerve inflammation^12,13^, suggesting that axonal damage and cell loss are induced by optic nerve inflammation.

Matrine (MAT), a natural quinolizidine alkaloid compound extracted from the herb root of Sophorae flavescens, with a molecular weight (MW) of 258.43 (C_15_H_24_N_2_O)^14–16^, is known for its various effects in animal models of EAE, including protection against apoptosis, tumor and fibrotic tissue development, and inflammation^17^. We have recently shown that MAT can ameliorate clinical signs and alleviate neuro-axonal injury in the CNS of EAE animals by regulatory T cells, reducing Th1 and Th17 cells in the CNS and periphery^18–20^, protecting the blood–brain barrier (BBB) from inflammatory attacks^21^, and increasing t he number of neural protective molecules^14,22,23^. However, the ability of MAT to suppress ON and protect RGCs has not been studied.

In the present study we tested our hypothesis that MAT can not only inhibit proinflammatory response, but also promote RGC survival by protecting these cells from inflammation-induced apoptosis. By using experimental ON in an EAE rat model, we examined the effect of MAT on inflammatory cell infiltration, demyelination, and neurodegeneration and RGCs apoptosis of the optic nerve, and the molecular mechanism underlying its therapeutic benefits has also been addressed.

## Results

### MAT treatment alleviated ongoing EAE in Wistar rats

**Figure 1:**
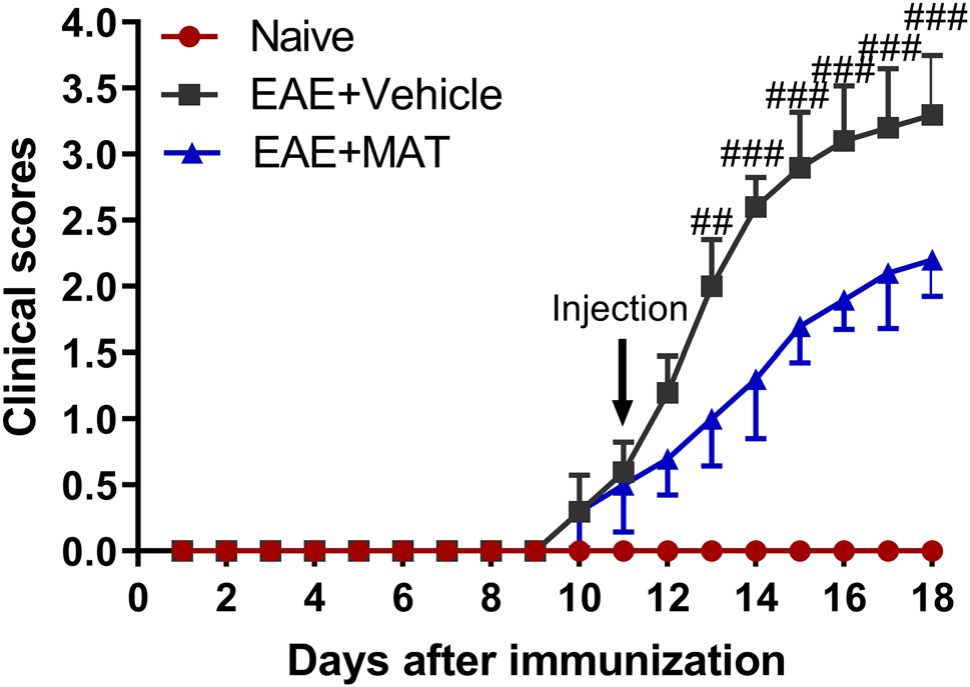
MAT ameliorated clinical signs of EAE. Wistar rats were immunized with spinal cord homogenate of guinea pig in CFA. Rats received MAT (250 mg/kg in 1 ml normal saline daily, i.p.) at onset of clinical signs of EAE until day 18 p.i., and control rats received the same volume of saline. All rats were evaluated daily for clinical scores of EAE in a blinded fashion from day 0 to 18 p.i. Clinical score—time graph of every group based on a 0–5 scale. Data represent mean ± SD (n = 10 rats per group). Clinical EAE scores were analyzed at single time points between treated and untreated rats using GraphPad Prism 5.0; (GraphPad Software). ^##^*P* < 0.01, ^###^*P* < 0.001, comparisons between vehicle- and MAT-treated groups.

As shown in Fig. 1, clinical signs of EAE began on day 10 p.i., and the MAT treatment was started on day 11 p.i. The clinical scores of MAT-treated rats were significantly decreased compared with the vehicle-treated rats, starting from day 13 p.i. up to day 18 p.i. (end of the experiment) when the scores were analyzed each individual day. These results confirmed the effect of MAT treatment in ongoing EAE.

### MAT treatment reduced optic nerve inflammation

**Figure 2:**
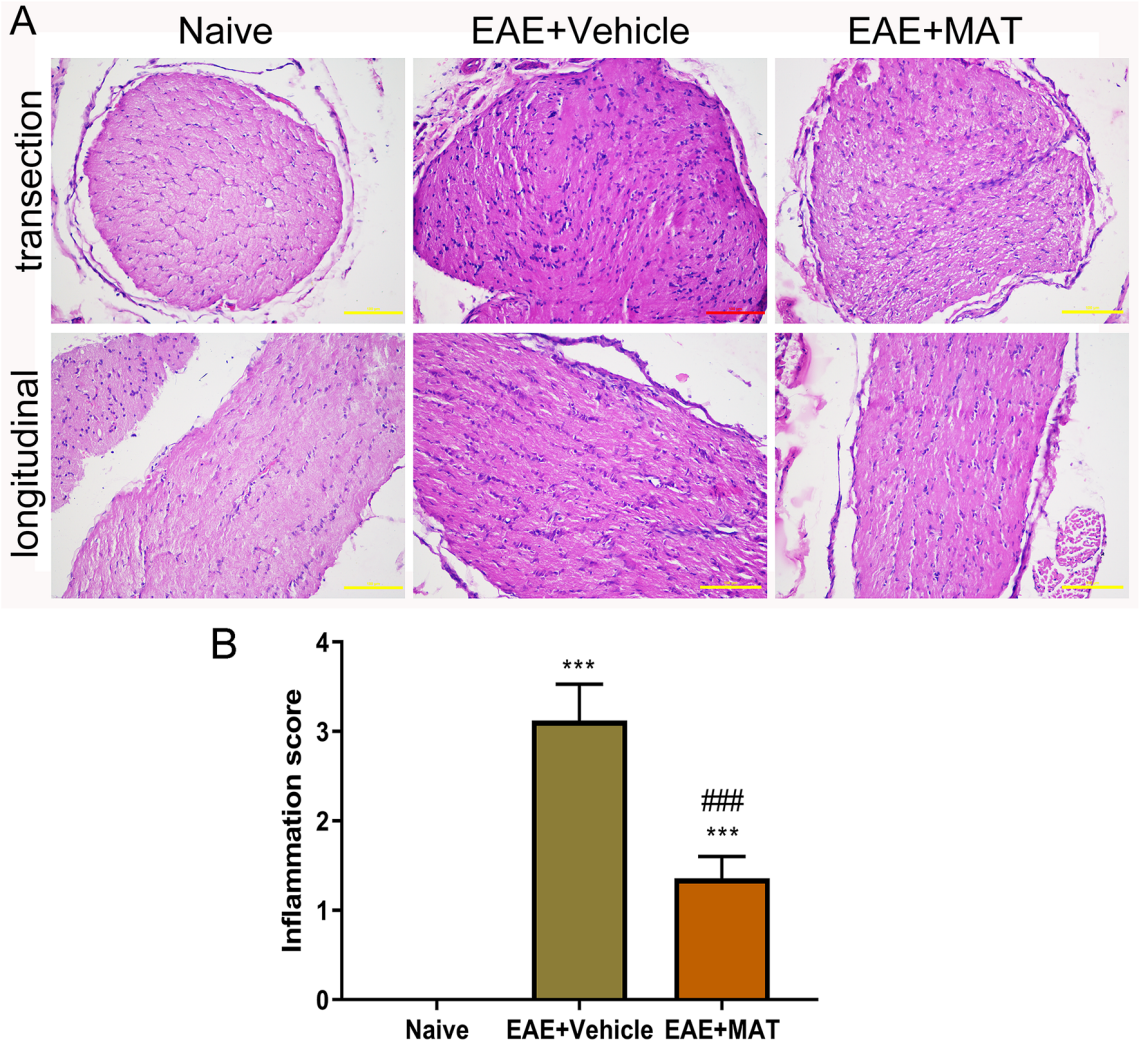
MAT attenuated the severity of optic nerve inflammation. (**A**) All rats described in Fig. 1 were euthanized and both sides of optic nerves were isolated and stained by H&E in transverse (upper row) and longitudinal (lower row) sections of optic nerves. Images were collected under the bright-field setting. Scale bars = 100 µm. (**B**) Degree of inflammatory cell infiltration in optic nerves. All results are expressed as mean ± SD (n = 40 per group: both transverse and longitudinal sections, both sides of optic nerves from 10 rats per group; 2 × 2 × 10 = 40 each group). Multiple comparisons were performed using one-way ANOVA, followed by Student–Newman–Keuls test. ***P < 0.001, comparison with the naive group. ^###^*P* < 0.001, comparisons between vehicle- and MAT-treated groups.

**Figure 3:**
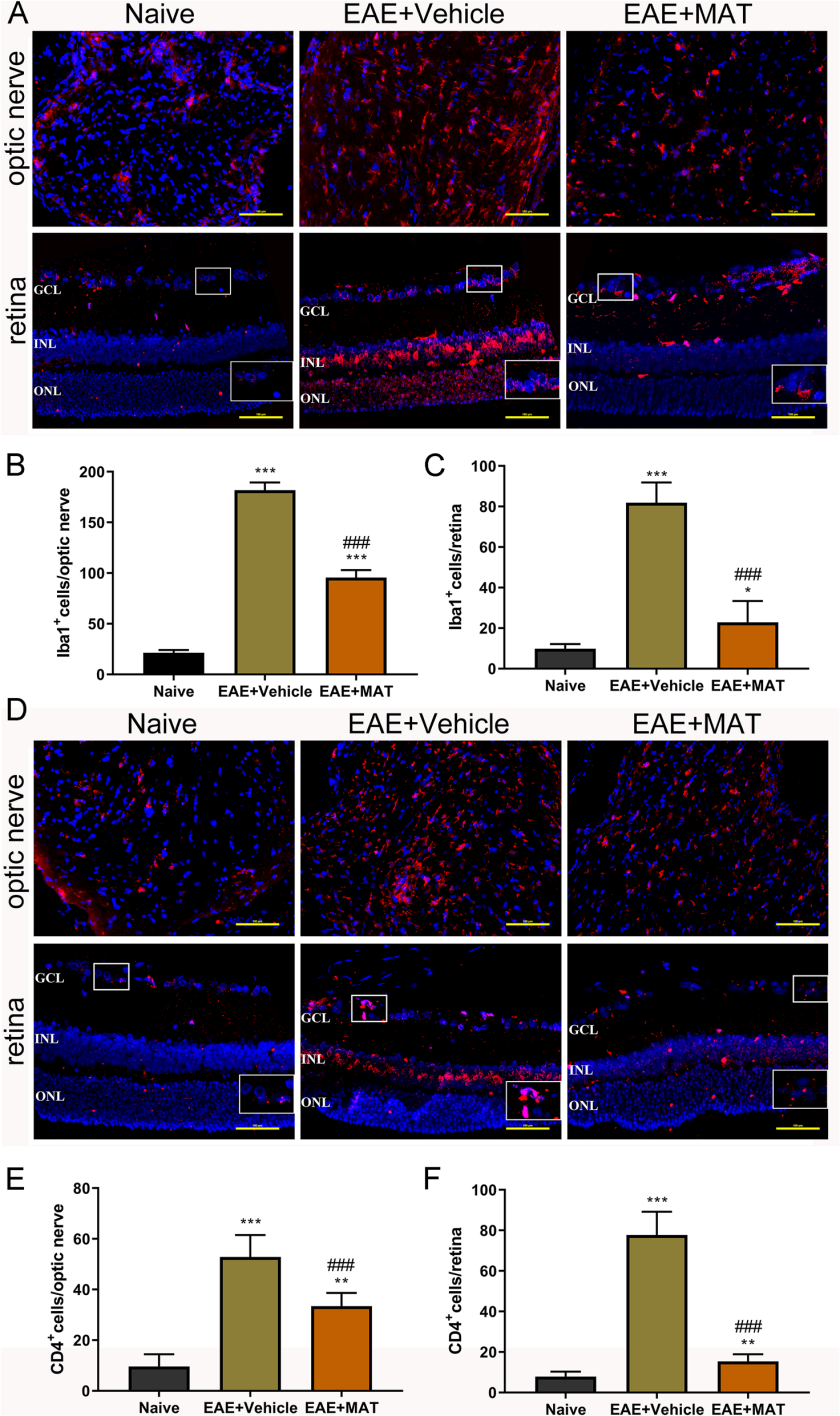
MAT treatment reduced numbers of Iba1^+^ and CD4^+^ cells in the optic nerve and the retina. Both sides of optic nerves and the retina were harvested from rats described in Fig. 1 at day 18 p.i. Immunofluorescence staining for Iba1 (**A**, red) and CD4 (**D**, red) in optic nerve and the retina sections. Scale bars = 100 µm. (**B**) and (**C**) Quantitative analysis of the number of Iba1^+^ cells in the optic nerve and retina, and (**E**) and (**F**) quantitative analysis of the number of CD4^+^ cells in the optic nerve and retina. ONL, outer nuclear layer; INL: inner nuclear layer; GCL, ganglion cell layer. Data are expressed as mean ± SD (n = 20 optic nerves and the retinas from 10 rats per group). Multiple comparisons were performed using one-way ANOVA, followed by Student–Newman–Keuls test. *P < 0.05, **P < 0.01, ***P < 0.001, comparison with the naive group. ^###^*P* < 0.001, comparisons between vehicle- and MAT-treated groups.

Consistent with the clinical scores, rats with clinical signs had massive inflammatory infiltration in the optic nerve of vehicle-treated rats, while those without clinical EAE signs also exhibited to a certain extent optic nerve inflammation. This infiltration was significantly decreased by MAT treatment (Fig. 2). The number of Iba1^+^ cells (macrophage/microglia) in the optic nerve and the retina was largely increased in immunized rats compared to naïve rats; MAT-treated rats had a significantly reduced number of Iba1^+^ cells when compared to the vehicle-treated group (Fig. 3A–C). A similar pattern was observed in CD4^+^ T cells, for which a significant reduction was observed after MAT treatment compared with vehicle treatment (Fig. 3D–F). These results indicate that MAT has a potent therapeutic effect in optic nerve inflammation.

### MAT treatment decreased optic nerve demyelination

**Figure 4:**
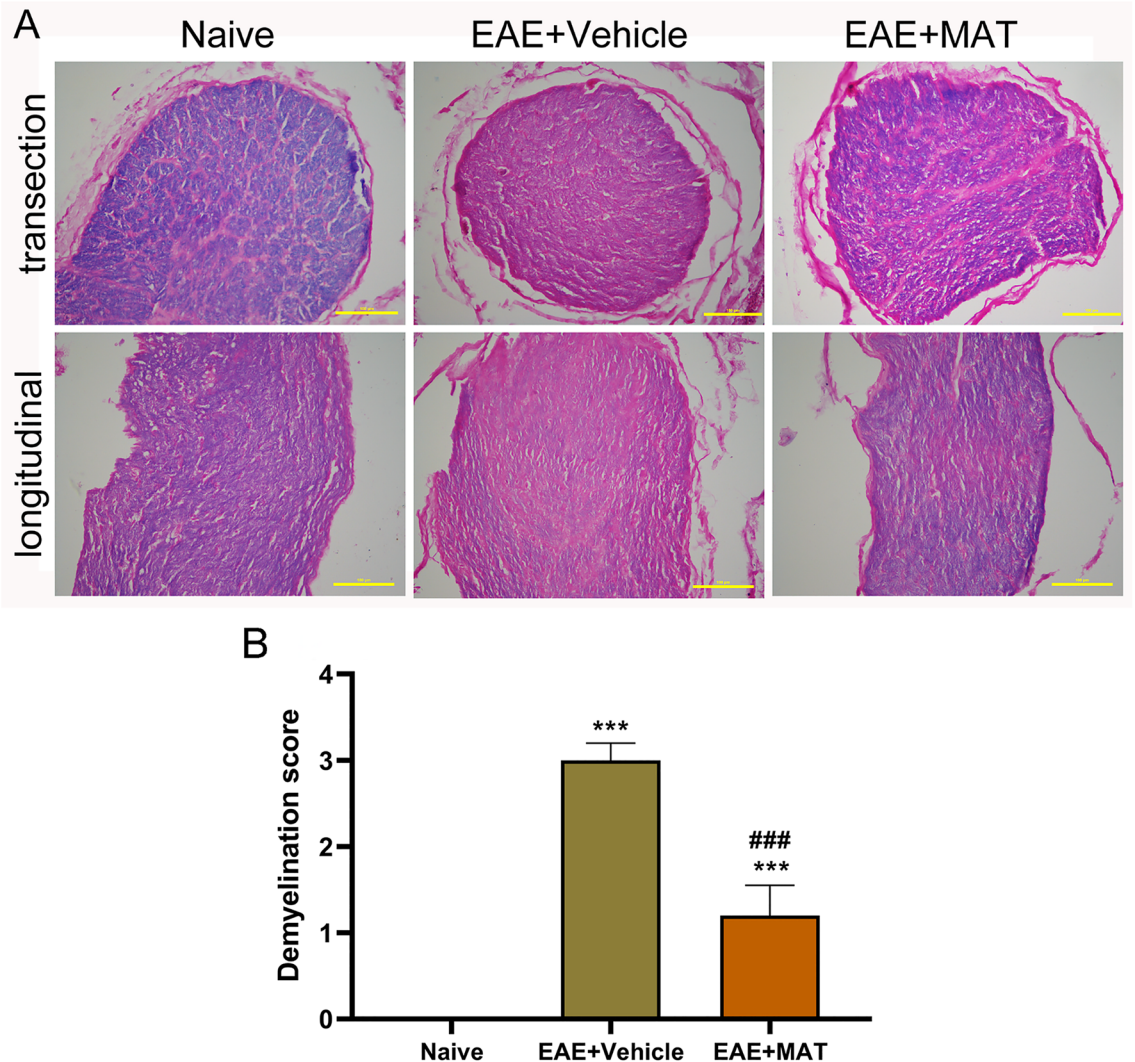
MAT attenuated demyelination in the optic nerve (**A**) To confirm whether MAT can protect the optic nerve from demyelination, both sides of optic nerves were isolated from rats in Fig. 1 and stained with Luxol fast blue (LFB), which stains myelin (**A**). Scale bars = 100 µm. (**B**) Mean scores of demyelination. For demyelination: 0, none; 1, rare foci; 2, a few areas of demyelination; and 3, large (confluent) areas of demyelination. All results are expressed as mean ± SD (n = 40 per group: both transverse and longitudinal sections, both sides of optic nerves from 10 rats per group; 2 × 2 × 10 = 40 each group). Multiple comparisons were performed using one-way ANOVA, followed by Student–Newman–Keuls test. ***P < 0.001, comparison with the naive group. ^###^*P* < 0.001, comparisons between vehicle- and MAT-treated groups.

To assess demyelination of the optic nerve after MAT treatment, all these nerves were assayed by LFB staining as described^12,24^. Optic nerves of both vehicle- and MAT-treated rats displayed significantly reduced myelin staining compared with that of naïve animals, and demyelination was markedly decreased after MAT treatment compared to vehicle-treated rats (Fig. 4A, B). Thus, MAT treatment can effectively mitigate demyelination in the optic nerves of diseased rats.

### MAT treatment reduced axonal loss in the optic nerve and retina

**Figure 5:**
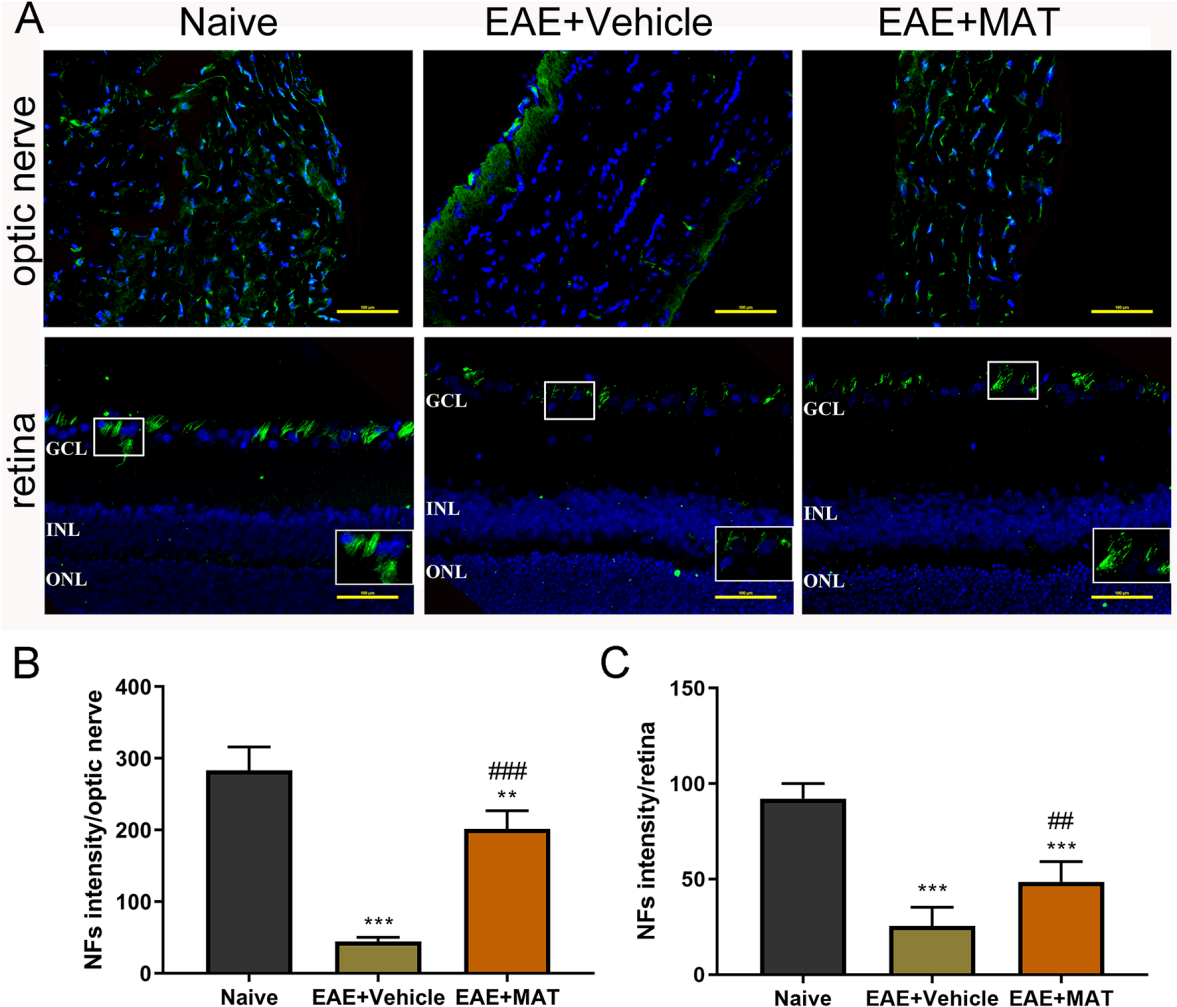
Effects of MAT on axonal loss in optic nerve. Both sides of optic nerves and the retina were harvested from naive and MAT- or vehicle-treated rats. (**A**) Detection of NF by immunofluorescence staining in the optic nerve and the retina. ONL, outer nuclear layer; INL: inner nuclear layer; GCL, ganglion cell layer. Scale bars = 100 µm. Quantitative analyses of immunofluorescence in optic nerve (**B**) and the retina (**C**) were expressed by average optical density (AOD) of NFs. Data are expressed as mean ± SD (n = 20 optic nerves and the retinas from 10 rats per group). Multiple comparisons were performed using one-way ANOVA, followed by Student–Newman–Keuls test. **P < 0.01, ***P < 0.001, comparison with the naive group. ^##^*P* < 0.01, ^###^*P* < 0.001, comparisons between vehicle- and MAT-treated groups.

Neurofilaments (NFs), a major component of the neuronal cytoskeleton, are believed to function primarily to provide structural support for the axon and to regulate axon diameter^25,26^. When optic nerve and retina sections of all rats were therefore stained with the NF antibody (Fig. 5A), our results showed that NF expression was significantly decreased in optic nerves of immunized rats compared with naïve ones, while this expression was markedly increased after MAT treatment (Fig. 5B, C).

### MAT treatment reduced RGC apoptosis

**Figure 6:**
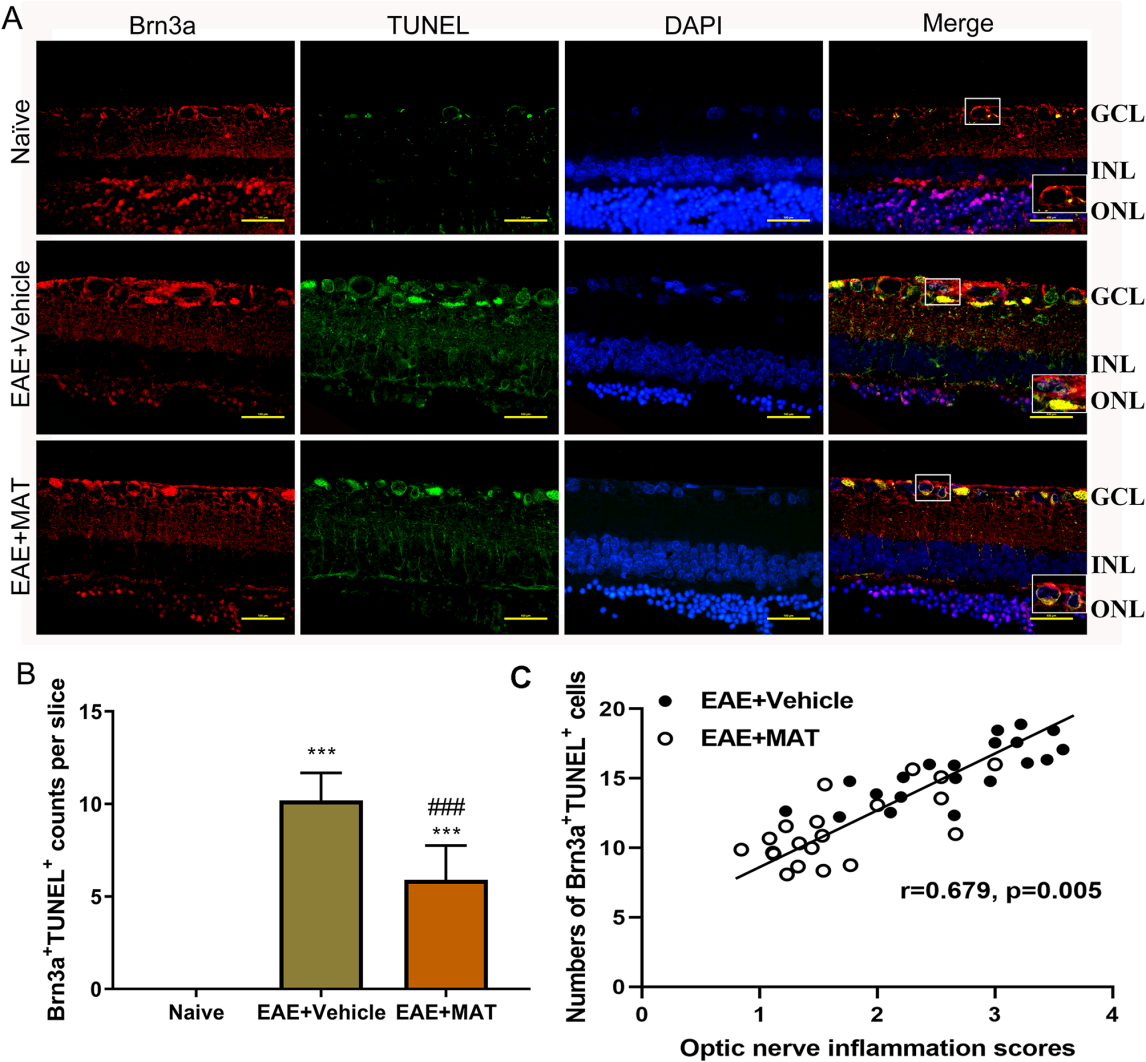
MAT treatment protected RGCs from apoptosis. Neuroprotective effects of MAT were evaluated by counting RGCs immune-labeled with Brn3a antibody and estimating the number of RGC deaths through TUNEL. (**A**) RGCs in the both sides of temporal retina were examined by immunofluorescent double staining by anti-Brn3a (red) and TUNEL (green), and all cells were co-stained with DAPI (blue). ONL, outer nuclear layer; INL: inner nuclear layer; GCL, ganglion cell layer. Scale bars = 100 µm. (**B**) Quantitative analysis for the numbers of apoptotic RGCs (TUNEL^+^ Brn3a^+^DAPI^+^). (**C**) Scatter plots between optic nerve inflammation scores and numbers of Brn3a^+^TUNEL^+^ cells showing significant positive correlation. Numbers of Brn3a + TUNEL + cells were compared between EAE + Vehicle and EAE + MAT groups using generalized estimating equation (GEE) models with optic nerve inflammation scores as a covariate to adjust for within-subject inter-eye correlations. Subsequently, an adjusted *P* value was used for multiple comparison according to the Bonferroni correction methods. ***P < 0.001, comparison with the naive group. ^###^*P* < 0.001, comparisons between vehicle- and MAT-treated groups.

We then determined whether MAT has an effect in protecting RGCs from apoptosis by double staining of anti-Brn3a (for RGCs) and TUNEL (for apoptosis) in the retina (Fig. 6A). No Brn3a^+^TUNEL^+^ apoptotic RCGs were found in naïve rats, while the number of these cells was increased in immunized rats. There was a significant decrease after MAT treatment compared with vehicle-treated rats (Fig. 6B). There was significant positive correlation between the optic nerve inflammation scores and numbers of Brn3a^+^TUNEL^+^ cells (Fig. 6C). An adjusted *P* value was used for multiple comparison according to the Bonferroni correction methods. These results indicated that MAT treatment can reduce RGC apoptosis, and thus promote their survival.

### MAT treatment promoted the shift of the Bcl-2/Bax ratio back towards antiapoptotic and promoted Akt phosphorylation in the retina

**Figure 7:**
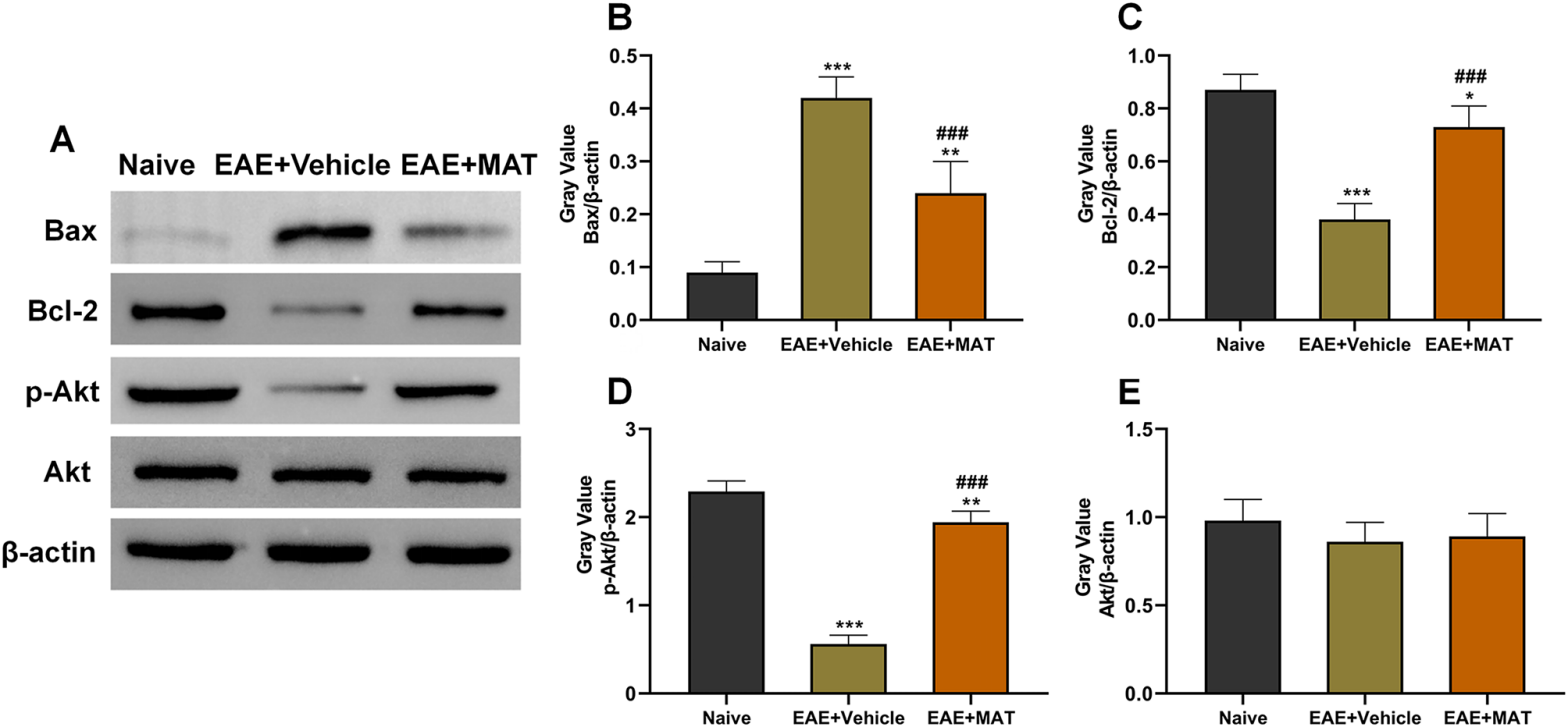
MAT treatment promoted the shift of the Bcl-2/Bax ratio back towards the antiapoptotic and Akt phosphorylation. The retinas were harvested from naive and MAT- or vehicle-treated rats. (**A**) Protein expression of Bax, Bcl-2, p-Akt and Akt was determined by Western blot. Ratio of gray value of Bax (**B**), Bcl-2 (**C**), p-Akt (**D**), Akt (**E**) to β-actin was calculated. Data represent mean ± SD (n = 5 per group). Multiple comparisons were performed using one-way ANOVA, followed by the Student–Newman–Keuls test. *P < 0.05, **P < 0.01, ***P < 0.001, comparison with the naive group. ^###^*P* < 0.001, comparisons between vehicle- and MAT-treated groups.

After we had documented that MAT treatment can reduce RGC apoptosis, we investigated the involved pro- and antiapoptotic intracellular signal transduction cascades. The Bcl-2 family is a well-known group of apoptosis-related proteins, and a shift in the expression of the Bcl-2 family of proteins to a more proapoptotic ratio in RGCs was observed during development of EAE^27^. For detection of the levels of proapoptotic member Bax and the antiapoptotic protein Bcl-2, we used Western blot to measure their protein levels in the retina (Fig. 7A). A higher level of Bax was detected in the vehicle-treated EAE rats, and MAT treatment significantly inhibited its expression (Fig. 7B). In contrast, the expression of Bcl-2 was significantly increased in the MAT-treated EAE group (Fig. 7C).

We then investigated another signal transduction pathway, Akt, which plays a major role in RGC apoptosis^27^, by determining the expression of p-Akt and Akt in the retina by Western blot (Fig. 7A). Compared with the vehicle-treated EAE group, the expression level of p-Akt, an activated form of Akt, was significantly increased in the retinas of the MAT-treated EAE group (Fig. 7D). There was no significant difference in the expression of Akt among all groups (Fig. 7E). Together, these results show that MAT inhibited the RGC apoptosis, likely by promoting the shift of the Bcl-2/Bax ratio back towards antiapoptotic and promoting Akt phosphorylation.

## Discussion

ON is characterized by inflammatory demyelination and axonal injury in the optic nerve, leading to RGC loss and visual dysfunction^28^. ON commonly occurs in MS patients and in its animal model, EAE, as well^8^. Previous studies have described the histopathological aspects of ON, but neuronal loss in animal models of experimental ON has been less well studied. It has been found that ON is not only an inflammatory condition, but also involves significant neurodegeneration^29^; however, few therapies are known to be effective for RCG protection, and neuronal loss in animal models of experimental ON has not been well addressed. We have in previous studies shown that treatment with MAT could suppress the development of EAE^22,30^, however, whether this natural alkaloid can protect neurons in ON is still unknown. Here we have for the first time provided evidence that MAT treatment resulted in clinical improvement in ON during EAE, as indicated by reduced inflammation and demyelination in the optic nerve^31^. The upregulated expression of neurofilaments and reduced RGC apoptosis after MAT treatment indicates that MAT may also have neuroprotective properties.

It has been shown that inflammatory responses play an important role in the development of ON^31^, and optic nerve demyelination and infiltration have also been found to correlate with the severity of clinical disease in EAE mice^32^. Among inflammatory cells, activated macrophages and microglia are the major cell types in ON that are closely associated with demyelination, axonal damage and loss of visual function^33^. Indeed, when an inflammatory event occurs, such as autoimmunity, neural injuries or ischemia, microglia rapidly become activated and begin migrating to the event site while releasing pro-inflammatory substances such as TNF-α and interleukins that lead to tissue damage^34^. Significantly more microglia have also been found in retinas of ON, which could be a direct response to RGC degeneration^35^. T cells, by secreting proinflammatory cytokines, play a major role in the inflammatory demyelination of the optic nerve^36–38^. Our data show increased numbers of CD4^+^ T cells and Iba1^+^ microglia/macrophages in the optic nerves of ON rats, which were significantly reduced after MAT treatment. The observation in the present study on the anti-inflammatory effects of MAT in experimental ON is consistent with findings in a variety of other inflammatory diseases and animal models. MAT possesses significant anti-hepatitis, immunosuppressive, anti-tumor, and anti-hepatic fibrosis capacities^39^. Previous research has shown that MAT can inhibit immune activities of T cells, B cells and macrophages, at relatively low doses, and it is known to have partially suppressed development of EAE^18^. In addition, MAT therapy significantly suppressed the production of proinflammatory cytokines, such as IFN-γ, TNF-α and IL-17, and blocked the migration of peripheral immune cells into the CNS^19^, suggesting that it may be beneficial in ON. Further, we have previously found that MAT inhibited BBB disruption in EAE, as shown by reduced Evans Blue extravasation, increased expression of collagen IV, ZO-1, and TIMP-1/-2, and reduced MMP-9/-2^21^. This effect would be an important mechanism for the reduced inflammation in optic nerves and the protective effect of MAT for RGCs.

NFs, which are synthesized in the neuron body and then transported into the axons, play a key role in the axonal cytoskeleton^40^. Our present study suggests that an effective treatment such as MAT can preserve this axon-associated protein from inflammation-induced damage. Consistent with our observations, phosphorylated neurofilament heavy chain was found increased in serum in an ON model of MOG-specific TCR transgenic mice, indicating the NFs were released into the bloodstream from damage to optic nerve axons^41^. Indeed, a threefold reduction in NF levels was shown in the pooled optic nerve samples from both eyes of these ON mice, which is consistent with reduced visual function and optic nerve atrophy visualized by MRI^42^. Similarly, a reduced level of NF expression was observed in the spinal cord of untreated EAE mice, while this level was significantly increased after treatment, accompanied by improved clinical score of disease^43^. On the other hand, demyelination of axons in the optic nerve results in apoptosis of RGCs, which is the major cause of vision loss in ON^8,12,38^, and inhibition of proinflammatory signaling resulted in a nearly complete prevention of axonal demyelination, as well as a drastic attenuation of RGC death in ON^44^. Consistent with these observations, we detected a large number of apoptotic RGCs in untreated rats, and the number was significantly reduced upon MAT treatment. These results, together with enhanced expression of NFs, suggest that MAT treatment reduces axonal loss, and then promotes RGC survival during experimental ON.

Two distinct intracellular pathways are involved in RGC apoptosis: a shift towards a more proapoptotic ratio in the Bcl-2 family and a down-regulation of p-Akt. The involvement of both these intracellular signal transduction cascades in RGC death following mechanical lesion of the ON has been demonstrated previously^27^. In the present study, we found that MAT treatment promoted the shift of the Bcl-2/Bax ratio back towards the antiapoptotic in RGCs. In addition, we showed that a strong reduction in p-Akt protein levels was detectable in the vehicle-treated EAE group, and that MAT treatment significantly promoted its expression. Thus, the inhibited RGC apoptosis would most likely result from shifting the Bcl-2/Bax ratio back towards antiapoptotic and promoting Akt phosphorylation.

It has been recently found that intranasal delivery could be an easy and effective approach for treatment of EON^45–47^ and EAE^48–50^. With the intranasal route, chemical drugs, peptides, viruses, plasmids and even cells can be successfully delivered to the CNS by bypassing the BBB^51^, and intranasal administration of a medication also accumulated at high levels in the optic nerve and vitreous^46^. Further, intranasal administration increased the efficacy of treatment compared to subcutaneous injection when the same dose was given in EAE^48^ or a greatly reduced treatment dose (e.g. 8 times less) with comparable effect when compared with i.p. administration in EAE^49,52^. Based on these interesting observations, we plan to test the effect of intranasal administration of MAT in EAE mice, investigating the possibility of increasing its effect and reducing the dose.

In summary, our study demonstrates that MAT effectively suppresses CNS inflammation, demyelination and axonal loss in optic nerves, as well as RGC apoptosis in experimental ON. The mechanisms underlying these effects may include: (1) its anti-inflammatory effects^18,19,38^; (2) the potential to promote production of neurotrophic factors, such as NGF and BDNF, as previously reported^14,15^. These effects, together, could convert a hostile environment into a supportive one for neural cells, thus reducing myelin and axonal damage of the optic nerve and protecting RGCs from apoptosis. Taken as a whole, MAT attenuated inflammation, demyelination and axonal loss in the optic nerve, and protected RGCs from inflammation-induced cell death. MAT may therefore have potential as a novel treatment for ON.

## Materials and methods

### Animals

Thirty female Wistar rats, 8–10 weeks of age, were purchased from the Beijing Vital-River Experimental Animal Company, China, and housed in specific pathogen-free conditions at the Henan Province Chinese Medicine Research Institute. Every effort was made to ensure minimal animal suffering, and the guidelines of the Animal Care and Use Committee of the Henan Province Chinese Medicine Research Institute were followed for all the procedures in this study. All animal experiments were performed in accordance with the ARVO Statement for the Use of Animals in Ophthalmic and Vision Research.

### Induction of rat EAE model

EAE was induced in 20 rats as described previously^53^. Briefly, guinea pig spinal cord homogenate (GPSCH) was made from an equal amount of guinea pig (Beijing Vital River Experimental Animal Company) spinal cord and pre-chilled saline, and then emulsified with the same volume of complete Freund’s adjuvant (CFA) (Sigma, St. Louis, MI, USA) containing 6 mg/ml Bacillus Calmette–Guérin vaccine (Solarbio Bio-Technology Co., Shanghai, China). Each rat was subcutaneously injected at four separate sites with 0.5 ml of antigen emulsion at the same day. All the experiments were approved by the Bioethics Committee of Zhengzhou University.

### MAT treatment and disease assessment

Immunized rats were randomly divided into two groups (n = 10 each group). The EAE incidence was 80%, and each EAE group contained two rats that did not show clinical signs of EAE. Treatment groups include: (1) MAT (MW: 264.36, a small molecule that was purchased from Chia-Tai Tianqing Pharmaceutical Co.), was injected intraperitoneally (i.p.) at 250 mg/kg in 1 ml normal saline daily, starting from day 11 post immunization (p.i.) until the end of the experiment (day 18 p.i.); (2) immunized rats that received the same amount of saline via i.p. served as control; (3) non-immunized naïve rats that received the same amount of saline i.p. served as naïve control. All rats were monitored and scored daily from day 0 to 18 p.i. by two independent observers in a blinded manner following the standard 0–5 EAE grading scale as previously published^6,8^: 0, natural; 0.5, partial tail paralysis;1, tail limpness or waddling gait; 1.5, loss of tail tonicity or waddling gait; 2, hind limb weakness; 2.5, partial limb paralysis; 3, paralysis of one limb; 3.5, paralysis of one limb and partial paralysis of another; 4,paralysis of two limbs; 4.5, moribund state, and 5, death.

### Histopathological evaluation of optic nerves

To assess the extent of CNS inflammation and demyelination, the rats were sacrificed on day 18 p.i. Rats were anesthetized by intraperitoneal injection of 1% pentobarbital sodium (50 mg/kg) and extensive perfusion with 0.9% normal saline. Both sides of the optic nerves were quickly harvested and post-fixed with 4% paraformaldehyde, embedded in paraffin, and cut into paraffin Sects. (2–5 µm). Cross-sectional optic nerve sections (the anterior part of the optic nerve) and longitudinal optic nerve sections (the posterior part of the optic nerve) were examined from both sides of eyes for H&E and LFB staining. The histological examination was performed and scored by light microscopy by two investigators in a blinded manner using a grading scale as previously published criteria^8^: 0, no inflammatory infiltration; 1, a few scattered inflammatory cells of the optic nerve or optic nerve sheath; 2, moderate inflammatory infiltrates; 3, severe inflammatory infiltrates; 4, massive inflammatory infiltrates. An Olympus BX53 microscope (Japan Olympus Corporation) was used for the histological examination. Scores of demyelination and inflammation were calculated by Image-Pro Plus 5.0 (IPP5.0) software.

### Immunofluorescence analysis of optic nerves and retina cross-sections

Both optic nerves and retinas from each rat were paraffin-embedded and cut into 5-μm-thick sections for immunofluorescence. CD4, Iba1 and neurofilaments (NFs) were assessed in the anterior part of the optic nerve and the retina, and Brn3a and TUNEL were done in the temporal retina. This area was used as a representative, given that, while most studies have tested entire retina, it has also been shown that the most significant changes occur in the temporal area^54^. Five optic nerves and retinal slices from each eye were used. First, non-specific binding was blocked with 3% bovine serum albumin (BSA) (Serotec, UK), and permeabilized with 0.3% Triton X-100 in 1% BSA-phosphate buffer saline (PBS) for 30 min. The optic nerve sections were incubated at 4 °C overnight with mouse anti-rat CD4 (1:100, Bioss, Beijing, China), mouse anti-rat Iba1 (1:100, Abcam, London, UK), rabbit anti-rat NFs (1:100, Bioss), followed by incubation with corresponding secondary antibodies (goat anti-mouse Cy3 conjugate, 1:200; goat anti-rabbit Alexa Fluor 488, 1:200; Invitrogen, Carlsbad, CA, USA) at room temperature for 2 h.

For double staining of RGCs and TUNEL (for apoptotic cells), comparable areas of the temporal retina were analyzed in all animals of all three groups. TUNEL reaction mixture was added before the primary antibody (Abcam) following the manufacture’s instructions. RGCs-positive were detected by rabbit anti-Brn3a (1:100, both from Abcam), followed by incubation with corresponding secondary antibodies—goat anti-rabbit Cy3 conjugate (1:200, Invitrogen, Carlsbad, CA, USA) for 2 h at room temperature. To assess the number of cells, a nuclear stain 4′,6-diamidino-2-phenylindole (DAPI, Roche, Shanghai, China), was added to tissue sections for 15 min prior to final washes after adding secondary antibodies. Finally, slides were visualized with confocal microscope (Olympus FluoviewFV1000).

Numbers of CD4^+^ and Iba1^+^cells that had been co-stained for DAPI were counted as positive cells. Brn3a^+^TUNEL^+^cells that were co-stained for DAPI were counted as apoptotic RGCs. The images were used for RGC counts with Image J (National Institutes of Health, USA) software, after image thresholding and manual exclusion of artifacts. Image J was also used to assess average optical density (AOD) of NFs. The anterior part of the optic nerve was used to quantify NF intensity. Exposure time was fixed for all microscope images. All these studies were performed by two investigators in a blinded manner.

### Western blot of retina

The retinas were lysed with ice-cold lysis buffer (1 × PBS, 1% Nonidet P-40, 0.5% sodium deoxycholate, and 0.1% SDS) supplemented with protease inhibitors and centrifuged. Supernatants were extracted, and protein concentrations were determined by BSA assay. Equal amounts of protein were separated by SDS-PAGE and then transferred onto a PVDF membrane. The membrane was blocked with 5% skim milk and incubated overnight at 4 °C with the primary antibody against Bax, Bcl-2 (both 1:200, Abcam), phospho-Akt (1:1000, Abcam), or Akt (1:1000, Abcam); membranes were washed in PBS-T and incubated with HRP-conjugated secondary antibodies against rabbit IgG (1:4000, Zymed Laboratories, USA). Labeled proteins were detected using the ECL-plus reagent (Servicebio, Wuhan, China).

### Statistical analysis

All the animal groups were coded and analyses were conducted by two researchers blind to experimental conditions. Multiple comparisons were performed using one-way ANOVA, followed by Student–Newman–Keuls test. Clinical EAE scores were compared at individual time points between vehicle- and MAT-treated rats. Statistical software (GraphPad Prism 5.0; GraphPad Software) was used for statistical analyses; *P* < 0.05 was considered significant. For all histological experiments, each eye was used as an independent data point, based on previous reports showing that optic neuritis can occur bilaterally, or unilaterally in either eye, and thus can occur as an independent event^55^. Numbers of Brn3a^+^TUNEL^+^ cells between EAE + Vehicle and EAE + MAT groups were compared using generalized estimating equation (GEE) models with optic nerve inflammation scores as a covariate to adjust for within-subject inter-eye correlations. Subsequently, an adjusted *P* value was used for multiple comparison according to the Bonferroni correction methods.

## Supplementary Information

Supplementary Information 1.

## Data Availability

The datasets generated for this study are available on request to the corresponding authors.
