# Supplementary material for: Matrine treatment reduces retinal ganglion cell apoptosis in experimental optic neuritis
Source: Sci Rep. 2021 May 4;11:9520. doi: 10.1038/s41598-021-89086-7 (PMC8097076; doi:10.1038/s41598-021-89086-7)

Supplementary Information

Matrine treatment reduces retinal ganglion cells apoptosis in experimental optic neuritis

Jian Kang[1,+], Shuqing Liu[1,+], Yifan Song[2], Yaojuan Chu[1], Mengru Wang[1], Yamin Shi[3], Fengyan Zhang[4,*] & Lin Zhu[1,*]

[1] Department of Pharmacy, The First Affiliated Hospital of Zhengzhou University, Zhengzhou, Henan, China

[2] Department of Ophthalmology, Peking University Third Hospital, Beijing Key Laboratory for Restoration of Injured Ocular Nerve, Peking University Third Hospital, Beijing, China

[3] Department of Chinese Medicine, The First Affiliated Hospital of Zhengzhou University, Zhengzhou, Henan, China

[4] Department of Ophthalmology, The First Affiliated Hospital of Zhengzhou University, Zhengzhou, Henan, China

* corresponding author: Lin Zhu, zhulin66zhulin@163.com

[+] these authors contributed equally to this work

Supplementary Figures

The original Western blot images

Figure 7 Bax

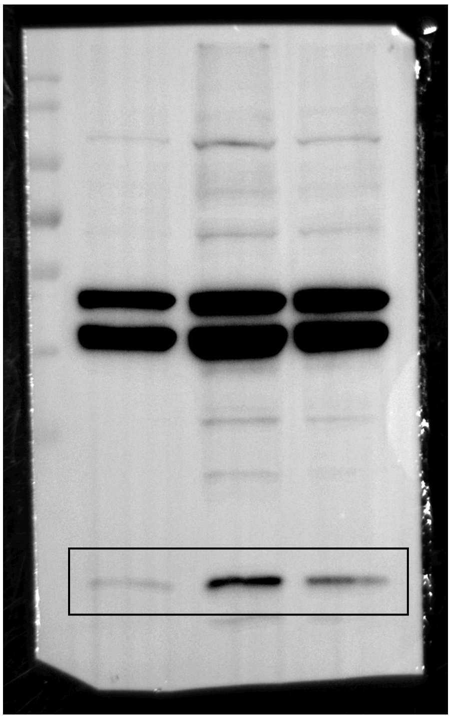

Figure 7 Bcl-2

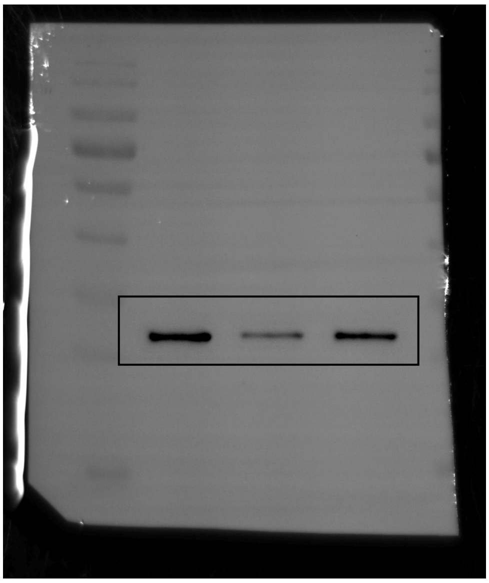

Figure 7 p-Akt

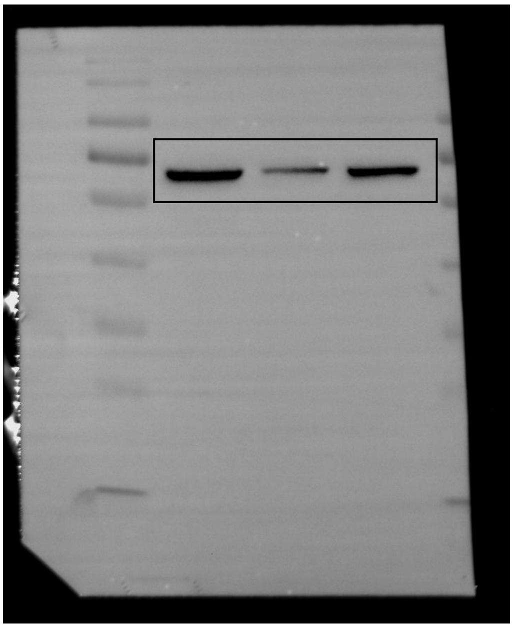

Figure 7 Akt

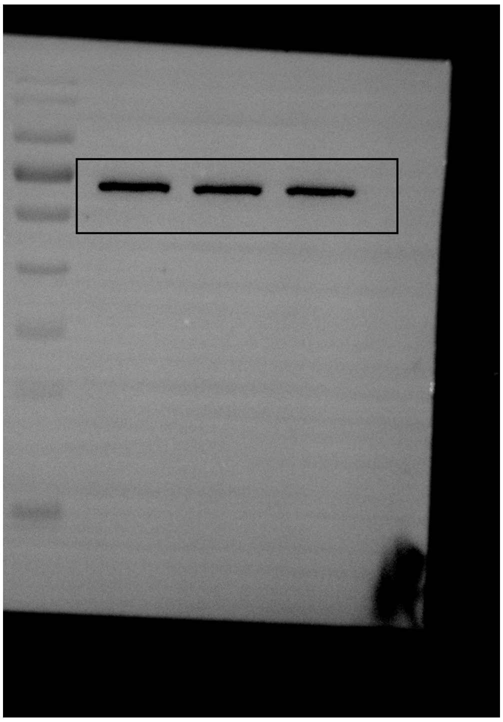

Figure 7 β-actin

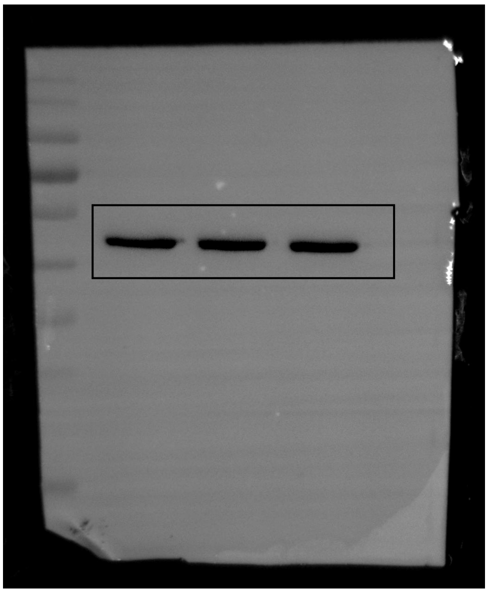

Supplement: Supplementary file 1 — Supplementary Information 1. [file 41598_2021_89086_MOESM1_ESM.pdf]
